# The Role of IgA in the Pathogenesis of IgA Nephropathy

**DOI:** 10.3390/ijms20246199

**Published:** 2019-12-09

**Authors:** Martina Perše, Željka Večerić-Haler

**Affiliations:** 1 Medical Experimental Centre, Institute of Pathology, Faculty of Medicine, University of Ljubljana, 1000 Ljubljana, Slovenia; 2 Department of Nephrology, University Medical Centre Ljubljana, 1000 Ljubljana, Slovenia; zeljka.vecerichaler@kclj.si; 3 Faculty of Medicine, University of Ljubljana, 1000 Ljubljana, Slovenia

**Keywords:** IgA, IgA nephropathy, mucosal immunity, immunoglobulin A

## Abstract

Immunoglobulin A (IgA) is the most abundant antibody isotype produced in humans, predominantly present in the mucosal areas where its main functions are the neutralization of toxins, prevention of microbial invasion across the mucosal epithelial barrier, and simultaneous maintenance of a physiologically indispensable symbiotic relationship with commensal bacteria. The process of IgA biosynthesis, interaction with receptors, and clearance can be disrupted in certain pathologies, like IgA nephropathy, which is the most common form of glomerulonephritis worldwide. This review summarizes the latest findings in the complex characteristics of the molecular structure and biological functions of IgA antibodies, offering an in-depth overview of recent advances in the understanding of biochemical, immunologic, and genetic factors important in the pathogenesis of IgA nephropathy.

## 1. Introduction

Immunoglobulin A nephropathy (IgAN) is the most frequently diagnosed type of glomerular disease in the world [1]. IgAN can be confirmed only by histopathological examination of the renal biopsy specimen, which reveals the presence of dominant or codominant mesangial deposits of immunoglobulin A (IgA) [2]. When IgAN is confined only to the kidney, it is diagnosed as a primary IgAN. However, IgAN could appear also as a secondary manifestation to a series of extra-renal conditions like chronic liver disease, diabetes, hypertension, amyloidosis, or lupus. In such cases, it is classified as secondary IgAN.

Primary IgAN remains the dominant form of primary glomerulopathies in adults, with a global prevalence of 2.5 cases per 100,000 adults per year [3]. Compelling data from biopsy and organ replacement registries suggest large geographic differences in IgAN distribution, with the highest incidence in East Asian populations (45 million people/year in Japan) followed by European (31 million people/year in France) and African populations [1]. However, there are some differences in the screening programs (particularly the indication criteria for renal biopsy) among countries across the world, which shed some doubts on the accuracy of these data. For instance, certain countries have an active urine testing program (i.e., regular checkups of population) and low threshold for kidney biopsy performance (isolated hematuria, proteinuria less than 1 gr/day, normal kidney function), while kidney biopsy practices in others include more strict criteria for kidney biopsy performance (proteinuria of higher grade and concomitant hypertension and/or reduced kidney function). Thus, the prevalence of IgAN is probably strongly underestimated, as not every patient with suspected kidney disease undergoes renal biopsy.

The hallmark of IgAN is the deposition of IgA in the glomeruli. Deposits are composed mainly of IgA, sometimes together with IgG or complement components such as C3. Biopsy specimens meeting these criteria have a range of histological changes that are reflected in the variable clinical course of IgAN [2]. The disease shows a broad spectrum of clinical presentations ranging from asymptomatic urinary abnormalities (like microscopic hematuria with or without proteinuria), to clinically overt kidney abnormalities (gross hematuria, nephrotic syndrome), and even to signs of progressive kidney failure. Some patients have a very mild form of the disease that requires little or no treatment, while others have progressive disease with up to 50% of patients developing end-stage renal disease within 20 years of diagnosis. The course of the disease is complex, the prognosis is variable, and the treatment is often only supportive. Nephrotic range proteinuria (exceeding 3 gr/day), hypertension, decreased glomerular filtration rate, and histological grading are robust predictors of adverse renal outcomes in IgAN. Primary IgAN patients with end-stage renal disease are candidates for kidney transplantation with questionable prognosis. Namely, IgAN has a strong tendency to recur in the kidney graft, which affects the outcome of the graft. It is important to note that data on recurrence rates of IgAN on kidney transplants vary due to differences in the screening programs (indication kidney graft biopsy vs. surveillance kidney graft biopsy). Results from indication kidney graft biopsy report that IgAN recurrence can occur in 13% to 50% of recipients [4], while results of surveillance biopsy report IgAN recurrence in up to 60% recipients [5] and in Asian populations even in 70% recipients [6].

Some factors have been recognized to present a risk for IgAN recurrence after transplantation. Those on the recipients’ side are younger age at diagnosis, male gender, higher degree of proteinuria, and a rapidly progressive course of IgAN before transplantation. Altered glycosylated IgA, soluble CD89 complexes [7], high serum IgA levels 6 months after transplantation [8], and serum IgG autoantibodies specific for galactose-deficient (Gd)-IgA1 recipients [9] have been associated with higher risk of IgAN recurrence. Moreover, latent IgA deposits in donor kidney are among frequently reported risk factors not only for IgAN recurrence, but also for delayed graft function and increased rejection rate of transplanted kidney [10]. However, it is noteworthy to mention that donor kidneys with IgA deposits when transplanted in non-IgAN recipients rapidly clear IgA deposits [5,11], which suggest that the basic cause of the disease is likely extra-renal.

The aim of the present paper is thus, firstly, to briefly summarize the complex biological role of IgA in the human body, including the receptors involved in IgA clearance and catabolism, and, secondly, to briefly highlight the complex mechanisms involved in the pathogenesis of IgAN and challenges to diagnose the primary IgAN.

## 2. The Biological Characteristics of IgA in Humans

**Figure 1:**
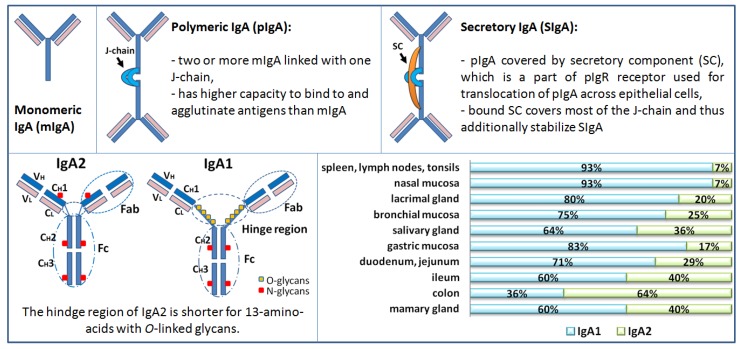
Schematic presentation of Immunoglobulin A (IgA) forms, subclasses, and distribution in humans. Distribution of IgA subclasses (IgA1 and IgA2) in external secretions of healthy adults. The relative proportions varying in different locations. Immunohistochemical studies and results from short- term culture experiments of human tissues supported the above-described distribution of the form of IgA (polymeric or monomeric) and the isotype (IgA1 or IgA2) in several fluids that parallels the distribution of cells in various tissues and organs. Measurements of antigen-specific antibodies in individual external secretions mirrored the distribution of IgA1- or IgA2- producing cells in the corresponding mucosal tissues.

IgA is the most abundant antibody isotype produced in humans, predominantly present in the mucosal areas (i.e., gastrointestinal, genitourinary, respiratory tract) but is also present in the blood [12]. In the human body, IgA can be found in three main forms: monomeric (mIgA), polymeric (mostly dimeric; pIgA), and secretory (SIgA) (Figure 1). Monomeric IgA can be found predominantly in the serum, SIgA in the mucosal areas and secretions, while pIgA can be found in serum but only in small amounts (Table 1). Polymeric IgA is usually dimer linked with joining J-chain (i.e., pIgA = IgA + J) produced by plasmablasts and plasma cells in the lamina propria of mucosae, where pIgA binds to the polymeric Ig receptor (pIgR) exposed on the basolateral side of secretory epithelial cells. After binding of pIgA to pIgR, the complex is endocytosed and transported across the cell by intracellular vesicular transport and excreted by exocytosis. In this process, a part of pIgR receptor termed secretory component (SC) is cleaved and pIgA linked with SC is released in the mucus layer as SIgA (i.e., SIgA = pIgA +SC) [13]. The main function of SIgA in mucosal areas and secretions is the neutralization of toxins, prevention of penetration and invasion of microbes (commensal and pathogens) across the mucosal epithelial barrier, thus preventing systemic infection while simultaneously maintaining a physiologically indispensable symbiotic relationship with commensal bacteria. The function of the serum IgA and antigenic stimulation for serum IgA is less clear. Serum concentration of IgA in humans is 1.0 to 2.0 mg/mL or 6%–15% of total serum Ig (in rats 0.1 mg/mL or less than 1% of total serum Ig) [12]. Characteristics of serum IgA and mucosal IgA are summarized in Table 1.

SIgA is more resistant to bacterial protease enzymes than mIgA or pIgA and it is well suited to function in protease-containing secretions; its enzymatic resistance seems to be enhanced when the antibody is complexed with antigen [13].

Humans have two subclasses of IgA antibody, IgA1 and IgA2, which are produced by plasmablast and plasma cells residing in the lamina propria of various mucosal and exocrine sites. Subclasses of IgA antibodies and plasmablasts are differently distributed in various body secretions. IgA1 predominate in serum, tears, saliva, nasal secretions, and small intestinal mucosa. The levels of IgA1 and IgA2 are comparable in vaginal secretions, while IgA2 predominates in the colon mucosa (Figure 1). It has been shown that the IgA subclass distribution depends on the nature of antigen [14]. IgA2 subclass is more stable than IgA1 [13]. Certain enteric and respiratory bacteria such as *Clostridium ramosum*, *Neisseria gonorrhea*, *N. meningitidis*, *Streptococcus pneumoniae*, and *Hemophilus influenzae* [15,16], and some oral bacteria involved in periodontal disease like *S. sanguinis* (previously *S. sanguis*), *S. mitior* (previously *S. mitis*), *Porphyromonas/Prevotella* (previously *Bacteroides*), and *Capnocytophaga* species produce enzymes that can selectively cleave SIgA1 in its extended (13-amino acid) hinge region [13,17,18].

## 3. Structure of Human IgA

IgA displays a T-shaped structure (other Ig display Y-shape) and is the most glycosylated form of antibody, with carbohydrates representing about 6% of its content. As mentioned, humans have two subclasses of IgA, IgA1 and IgA2, which differ structurally (Figure 1). The heavy chains of IgA1 molecules contain a 13-amino acid sequence in the hinge region segment between the Cα1 and Cα2 domains. This region has a high content of serine and threonine residues, which are the sites of attachment of up to nine potential *O*-glycans. Usually, three to six of these sites are *O*-glycosylated. *O*-glycans consist of *N*-acetylgalactosamine (GalNAc) linked with galactose (Gal; β1,3-linkage). The GalNAc-Gal disaccharide may be sialylated (linked with sialic acids) on GalNAc (α2,6-linkage), Gal (α2,3-linkage), or both [15,16,19].

IgA also contain *N*-glycosylation sites on each heavy chain, which are binding sites to Fc receptors on myeloid cells. The number and composition of *O*- and *N*-glycan chains can vary among individuals. *O*-linked glycans in IgA1 hinge region are involved in the binding to antigens or IgA receptors involved in clearance of serum IgA, while *N*-linked glycans in various IgA are involved in binding to Fc receptors, opsonization of antigens, and subsequent phagocytosis of IgA immune complexes. Thus, polymorphism of *O*- and *N*-linked glycosylation of IgA may affect clearance from circulation, removal of immune complexes, complement activation, and interaction with bacteria [16].

## 4. IgA Clearance and IgA Receptors

As mentioned above, the amount of IgA synthesized each day is much higher than the amounts of IgG (30 mg/kg BW) and IgM (8 mg/kg BW) together. It is important to note that in the circulation IgA is the second most abundant antibody (after IgG) [12]. Thus, to avoid deposition of IgA in the tissues, the clearance of the IgA from the circulation needs to be carefully regulated.

The clearance and catabolism of serum IgA has been investigated using radioactively labeled IgA. It was found that over 90% of serum IgA is cleared by internal metabolism with a half-life of 3–5 days. The majority of IgA (mIgA1, pIgA1, pIgA2) is taken up and catabolized in the liver, followed by the spleen and kidney [22]. Human hepatocytes express asialoglycoprotein receptors (ASGPR) that bind various serum asialoglycoproteins, including IgA [23]. This receptor recognizes GalNAc or Gal of *O*-linked glycans in the IgA1 hinge region and thus does not bind IgA2 or IgA1 linked with immune complexes (IgA-Ag). Another mechanism of serum IgA clearance is the reticuloendothelial system, particularly Kupffer cells that express Fc alpha receptor 1 (FcαRI also known as CD89) [12]. FcαRI is the best-characterized IgA-specific Fc receptor, which is constitutively expressed not only on Kupffer cells but also on other myeloid cells such as neutrophils, eosinophils, and monocytes. CD89 recognizes Fc domain of IgA and is involved in clearance of IgA linked with immune complexes.

A portion of serum IgA is also transported undegraded from the circulation into the bile [22]. Human liver expresses secretory component (pIgR) only on bile duct epithelial cells (rats also on hepatocytes). Thus, serum IgA in humans does not make significant contributions to IgA in gastrointestinal secretions [12].

It was known for a long time that IgA clearance takes place also in mesangial cells of the kidney, but the receptors involved in IgA clearance or IgA deposition were not identified. Recently, it was discovered that mesangial cells express at least two IgA receptors, i.e., transferrin receptors (TfR, CD71) [24] and β-1,4-galactosyltransferase1 (GalT1) receptor [25], which are responsible for clearance of IgA from the circulation.

In addition, the process of clearance of IgA linked with or without immune complexes takes place also in other cells, which express various specific and unspecific IgA receptors, such as Fcα/μ receptors [26], SC receptors [27], M cell IgA receptors [28], and FcγRI receptors [29]. Fc receptor-like (FcRL) proteins are a family of cellular receptors homologous to FcγRI and are predominantly expressed by B cells (ITAM/ITIM signaling) [29]. Receptors can interact with the Fc tail, carbohydrate side chains, or accessory molecules such as the J-chain or secretory component (SC). Receptors involved in IgA clearance and catabolism are summarized in Table 2. From Table 2, it can be seen that multiple IgA-specific receptors have been described, although the functions of several of these receptors are not yet completely clear. Therefore, to better understand IgAN or mechanisms involved in tissue deposition of IgA a better understanding of IgA clearance and receptors involved into IgA catabolism is needed.

## 5. IgA Deposits in Kidney

As already mentioned, IgA deposits in the glomeruli are the hallmark of IgAN. The diagnosis of primary IgAN therefore always requires a histopathological examination of the kidney biopsy specimen [2]. Since kidney biopsy is an invasive procedure associated with a substantial risk of complications, investigation of the prevalence of glomerular IgA deposition in a normal live population is thus not possible. However, the prevalence of glomerular IgA deposits in the general population can be estimated using data from kidney transplants (where kidney biopsy is an essential part of the procedure) or studies investigating control necropsy population that died from traumatic injuries, with no clinically apparent symptoms of renal disease or other IgA associated diseases. Unfortunately, such studies are scarce and rarely noticed, although the results are very valuable. Nevertheless, the reported prevalence of IgA deposits in asymptomatic individuals is summarized in Table 3. Strikingly, data from necropsy studies or kidney transplant studies revealed that 2.4% to 16% “healthy” individuals (i.e., without clinical symptoms or signs suggestive of renal abnormalities) may have IgA deposits in their kidneys. Thus, these studies clearly show that the current prevalence of primary IgAN is underestimated and that our current understanding on the role of IgA deposits in kidneys and the pathogenesis of IgAN can be limited.

## 6. Proposed Risk Factors in the Pathogenesis of IgAN

Various mechanisms have been proposed to be responsible for the IgA deposits in the mesangial cells, including increased production of Gd-IgA, decreased IgA clearance and IgA receptor alterations, and complement involvement, which are briefly described in the following sections.

### 6.1. Increased Production of Galactose-Deficient IgA1 (Gd-IgA1)

Numerous studies have shown increased proportion of Gal-deficient (Gd)-IgA1 in the serum of IgAN patients and proposed increased serum Gd-IgA1 levels as a risk factor for IgAN. However, it has been shown that the composition of *O*- glycans in the hinge region of serum IgA1 is usually heterogeneous. The commonest forms include GalNAc-Gal disaccharide without sialic acid, or in mono- or di-sialylated form [16,19]. Although a variant with terminal GalNAc or sialylated GalNAc can be observed in the serum of healthy people, it was found to be more common in the serum of IgAN patients [15].

**Figure 2:**
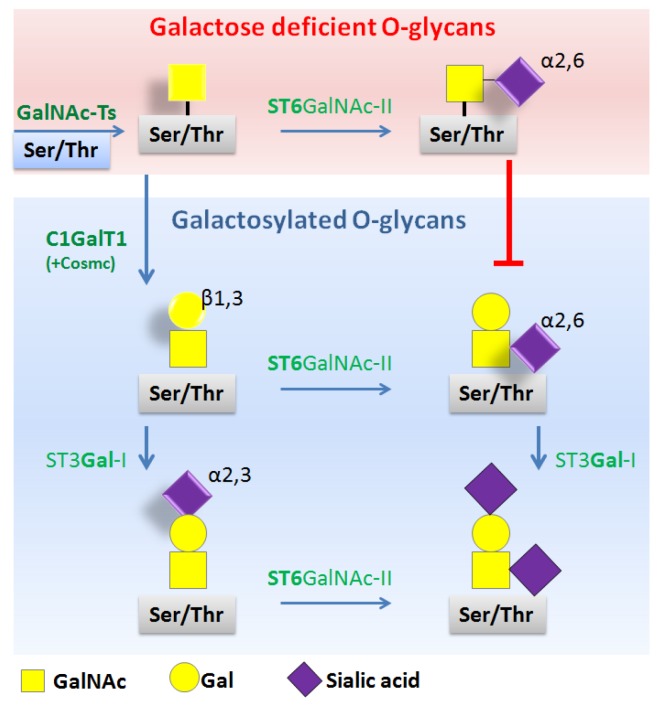
Pathways of IgA1 *O*-glycosylation in the hinge region. *O*-glycans are synthesized in a step-wise manner, beginning with the attachment of GalNAc to serine (Ser) or threonine (Thr) (catalyzed by GalNAc-Ts enzymes). Next step is the attachment of Gal (catalyzed by C1GalT1 enzyme in the presence of chaperone Cosmc), followed by the attachment of sialic acid by different enzymes: ST3Gal-1 for sialylation of Gal and ST6GalNAc-II for sialylation of GalNAc. If sialic acid is linked to GalNAc prior to attachment of Gal, it disables subsequent attachment of Gal and lead to galactose-deficient *O*-glycans. Symbol nomenclature for glycans: yellow square (GalNAc), yellow circle (Gal), purple diamond (N-acetylneuraminic acid = sialic acid).

To better understand the mechanisms involved in the production of Gd-IgA1, the process of *O*-glycosylation is briefly described. The hinge region of IgA1 is glycosylated post-transcriptionally in a step-wise manner. It begins with the attachment of GalNAc to the oxygen atom of serine (Ser) or threonine (Thr) (usually Thr225, Thr228, Ser230, Ser232, Thr233, Thr236) in the hinge region. Reaction is catalyzed by *N*-acetygalactosaminyl-transferases (GalNAc-Ts) (i.e., mainly GalNAc-T2 [42], less likely GalNAc-T1, GalNAc-T11 [43], or GalNAc-T14 [44]). The next step is the attachment of Gal to GalNAc, which is catalyzed by enzyme C1GalT1 (core 1 synthase, glycoprotein-*N*-acetylgalactosamine 3-β-galactosyltransferase) and chaperone C1GalT1C1 (also known as Cosmc, which is required for the stability of C1GalT1 enzyme) [45]. Sialic acid (*N*-acetylneuraminic acid) can be then added to each glycan by different enzymes, i.e., α2,3-sialyltransferase (ST3Gal-1) for sialylation of Gal and α2,6-sialyltransferase (ST6GalNAcII) for sialylation of GalNAc. However, when sialylation occurs on terminal GalNAc (without Gal), attached sialic acid prevents galactosylation (i.e., subsequent attachment of Gal) and results in Gal-deficient IgA1 as shown in Figure 2 [19].

In recent decades, numerous studies have shown that most IgAN patients have increased serum levels of Gd-IgA1 in comparison to healthy controls. Recently, heritable studies investigating serum Gd-IgA1 levels were performed. It was found that serum Gd-IgA1 levels were often elevated not only in patients with IgAN but also in 39% to 80% of their first-degree relatives [45,46,47]. Inheritance of high Gd-IgA1 serum levels has been found in a dominant pattern in all ethnic groups (European, Asian, African American) in adult IgAN patients [46,47,48], pediatric patients with IgAN and IgA vasculitis (previously termed Henoch–Schönlein purpura nephritis; HSPN) [49], and healthy monozygous and dizygous twins [50]. Increased levels of Gd-IgA1 were defined in relative terms, as levels that are 75% or 90% or 95% greater than the levels observed in geographically matched controls. A summary of heritability studies is shown in Table 4.

Interestingly, a recent systematic review analyzing the predictive value of serum Gd-IgA1 on risk of IgAN has reported similar findings. It found no significant differences in the level of serum Gd-IgA1 between IgAN patients and their first-degree relatives. However, the level of serum Gd-IgA1 was found significantly higher in IgAN patients than in healthy controls or patients with other renal diseases, but the level of serum Gd-IgA1 was not associated with the disease severity [51].

### 6.2. Genetic Factors Associated with Gd-IgA1 or IgAN

To identify common risk genes in large populations, several genome-wide association studies (GWAS) have been performed since 2010. GWAS have collectively identified more than 20 distinct loci associated with risk of IgAN (see Table 5).

It was recognized that identified loci encode multiple proteins that play a role in innate immunity against microbes (*DEFA, CARD9, ITGAM-ITGAX, VAV3*), complement activation (CFHR1/3, *ITGAM-ITGAX*), intestinal mucosal barrier maintenance, and regulation of mucosal IgA production (TNFSF13, LIF/OSM) and are thus also related with other immune-related diseases such as inflammatory bowel disease (Table 6) [52,53,54,55,57]. However, it was also recognized that currently identified genetic loci explain less than 8% of overall IgAN risk (Table 5). Interestingly, GWAS identified also some loci that were associated with genes involved in IgA1 posttranscriptional glycosylation such as *ST6GAL1* [56], *C1GALT1,* and *C1GALT1C1* [57]. However, it was reported that these loci may account for around 2% and 7% of variability in serum Gd-IgA1 of people living in East Asia and Europe, respectively [57]. In addition, a recent study showed that although *C1GALT1* genotype is associated with risk of IgAN, it explains only 3% of the variability in Gd-IgA1 levels, while variation at *C1GALT1C1* was not statistically significant [45].

### 6.3. Nongenetic Factors that Modify Glycosylation of IgA1

As mentioned above, the process of *O*-glycosylation of IgA1 hinge region is post-transcriptionally regulated. Differential *O*-glycosylation of IgA1 antibodies after antigen exposure at different induction sites is a known process. For instance, IgA1-*Helicobacter pylori* binding at mucosal surface result in Gd-IgA1, while IgA1-tetanus toxoid binding within systemic compartment result in heavily galactosylated IgA1. It is known that serum Gd-IgA1 level varies by age, and pediatric controls have lower levels of Gd-IgA than adults [47,49].

Thus, besides genetic factors, there are also epigenetic mechanisms such as microenvironment (cytokines) that affect *O*-glycosylation as a consequence of elevated transcription of the *ST6GALNAC2* gene encoding enzyme ST6GalNAcII responsible for sialylation, increased enzymatic activity of ST6GalNAcII [60], or decreased expression and activity of C1GalT1 and chaperone Cosmc, which result in the production of Gal-deficient IgA1 [61]. Namely, IL-6, and to a lesser degree also IL-4, was found to increase the production of Gd-IgA1 via modification of the activity of key enzymes involved in *O*-glycosylation, i.e., *C1GALT1*, *C1GALT1C1*, and *ST6GALNAC2*. IL-6 increases expression and activity of enzyme ST6GalNAc-II and decreases expression and activity of C1GalT1 and chaperone Cosmc, which results in the production of Gal-deficient IgA1 [61].

Nevertheless, increased levels of Gd-IgA1 in the serum or deposits of IgA1 or Gd-IgA1 in the kidney by themselves do not trigger clinically overt kidney disease. It is important to keep in mind that the blood relatives of IgAN patients had increased serum levels of Gd-IgA1 but no detectable kidney disease, indicating that additional factors are required for IgAN to develop [46,50]. In addition, in vitro studies have shown that proliferation of cultured mesangial cells is stimulated only in the presence of formed immune complexes. Since increased levels of IgA1 or Gd-IgA1 and Gd-IgA1 containing immune complexes were found in the serum of IgAN patients, together with prolonged half-life of Gd-IgA1 clearance, it was suggested that blood clearance may be delayed due to impaired catabolism by IgA receptors.

## 7. Decreased IgA Clearance and IgA Receptor Alterations

Among all above-mentioned receptors, FcαRI was one of the most investigated receptors. As already mentioned, FcαRI is a specific IgA receptor constitutively expressed in myeloid cells that specifically binds Fc domain of IgA. It can bind monomeric or polymeric IgA as well as IgA bound in immune complexes. It was found that interaction of monomeric serum IgA with FcαRI induces anti-inflammatory effects, while interaction of IgA immune complexes with FcαRI results in cross-linking and induction of proinflammatory responses. The bifunctional role of FcαRI is mediated by FcRγ adaptor, which contains an immunoreceptor tyrosine-based activation motif (ITAM) [62]. FcαRI expressed without FcRγ adaptor recycles monomeric IgA and thus plays an essential role in mIgA homeostasis, while FcαRI associated with FcRγ mediates either activating or inhibitory responses, which depends on the type of the ligand, i.e., multimers or monomers [62].

Cross-linking of FcαRI by IgA immune complexes (or IgA opsonized pathogens) induces a variety of processes, including phagocytosis, antibody-dependent cellular cytotoxicity, release of inflammatory mediators, and cytokines as well as antigen presentation, reactive oxygen species production, release of neutrophil chemoattractant leukotriene B4 (LTB4), and neutrophil extracellular traps (NETs) [63]. Binding of SIgA to FcαRI is hampered due to steric hindrance of SC, thus SIgA is not efficiently taken up by neutrophils or Kupffer cells. However, SIgA can trigger respiratory burst in neutrophils, although less efficiently compared to serum IgA.

However, mutation in FcαRI (Asn58 to Glu58) or removal of sialic acids, which alters glycosylation pattern of FcαRI, can importantly affect IgA binding affinity and IgA clearance. Alterations in IgA1 glycosylation and impaired sialylation of FcαRI were found to be associated with increased binding of IgA1 to FcαRI on neutrophils of IgAN patients. However, it was found that IgAN patients have markedly decreased FcαRI expression on blood phagocytic cells [64].

In addition, IgA clearance by FcαRI can be hampered due to other molecules that recognize a similar site on FcαRI as IgA such as pentraxins, the acute phase C-reactive protein, serum amyloid P component, and bacterial proteins. Some pathogens like *Staphylococcus aureus* or *Streptococci* produce several decoy proteins that obstruct binding of IgA to FcαRI, which represent an important evasion strategy for pathogens to escape IgA-mediated phagocytosis.

Serum Gd-IgA1 bound with immune complexes can be cleared by FcαRI (CD89) expressed on phagocytic cells (neutrophils, monocytes, Kupffer cells). However, in contrast to the increased circulating levels of IgA1-IgG complexes observed in severe IgAN patients, levels of IgA-sCD89 complexes were decreased, suggesting that CD89-containing complexes could be selectively trapped in the mesangium, aggravating the disease [62].

Overexpression of TfR1 in mesangial cells was found in patients with IgAN and celiac disease [24]. It was found that IgA1 immune complexes and Gd-IgA1 have a higher affinity for TfR (CD71), suggesting CD71 involvement into IgA deposition [62].

## 8. Four Hit Hypothesis and Other Hypotheses

Since increased levels of Gd-IgA1 and Gd-IgA1 containing immune complexes were found in the serum of IgAN patients, it was soon accepted that galactose residues on Gd-IgA1 are recognized as targets by Ig specific for GalNAc (mainly IgG2, less frequently IgA1 antibodies), which result in the formation of circulating immune complexes (Gd-IgA1-IgG2/IgA1) and mesangial deposition [65]. A situation analogous to the Tn syndrome (OMIM 300622) is a rare autoimmune disorder in which a Gd-membrane glycoprotein in blood cells of all lineages is recognized by naturally occurring antibodies.

The well-documented four hit hypothesis has been developed to explain the pathogenesis of IgAN. Hit 1 begins with the increased production of Gd-IgA1. Hit 2 involves formation of antiglycan antibodies, which specifically recognize the Gd-IgA1. Hit 3 involves formation of immune complexes between Gd-IgA1 and antiglycan-specific antibodies, which may activate the complement pathways. Hit 4 involves accumulation of these complexes in the glomerular mesangial cells, inducing proliferation and secretion of extracellular matrix, cytokines, and chemokines, which ultimately result in renal injury [65]. Proposed underlying molecular mechanisms in IgAN are excellently summarized elsewhere [66]. However, it is important to be aware that this hypothesis, including underlying molecular mechanisms, is mostly based on *in vitro* studies. Thus, the value of four hit hypothesis needs to be evaluated and confirmed in clinical practice.

### The Role of SIgA and Complement in IgAN

Nevertheless, a less known hypothesis suggests that SIgA may be importantly involved in the primary IgAN. It is widely known that mesangial IgA deposits are mostly classified as dimeric IgA, predominantly of pIgA1 and less frequently of pIgA2 subclass [67]. Interestingly, although glomerular deposition of SIgA in biopsies was reported in 15% of IgAN patients, these data have not been widely documented [68]. As explained above, SIgA is produced only at mucosal sites and therefore SIgA deposits suggest the involvement of mucosal immune response in IgAN. It is noteworthy that clinical studies report association between primary IgAN and mucosal infections, such as upper respiratory tract infections and especially tonsillitis [69]. In addition, pIgA is produced mainly at secretory sites, while serum mIgA is usually produced in bone marrow. Thus, it is likely that pIgA1 deposits in kidneys of IgAN patients are involved in the mucosal immune response. The role of SIgA and complement components is excellently explained by Oortwijn et al. [70]. Furthermore, a recent GWAS study revealed that genes involved in immunity against intestinal pathogens can be implicated in IgAN [55].

Recently, it was also recognized that complement components are also involved in pathogenesis of progressive forms of IgAN. As already mentioned, the diagnosis of primary IgAN is based on the presence of IgA as the predominant Ig deposit in glomeruli (mostly detected in the mesangium, sometimes additionally in capillary walls). In addition to IgA deposition, the biopsy can reveal glomerular IgG deposition, which is present in more than trace levels but less than IgA levels (reported in 15–85% of IgAN biopsy samples), complement component C3 (reported in less than 90% of biopsy samples), or C4d (reported in 30%–50% of IgAN biopsy samples) [2]. Importantly, the presence of IgG and/or complement component (C3 or C4d) has been associated with more aggressive clinical disease and subsequent development of end-stage renal disease [2]. An increasing numbers of studies suggest involvement of alternative and lectin pathways, which likely play an important role in amplifying the inflammatory response caused by formation of immune complexes and their deposition in the glomerular mesangial cells. The emerging role of complement proteins in the IgAN are excellently described elsewhere [71].

## 9. Biomarkers of IgAN

Currently, analysis of renal biopsies is essential for the diagnosis of IgAN. However, kidney biopsy is frequently not performed for several reasons, including invasiveness and risk of bleeding. Therefore, reliable biomarkers are desirable for the noninvasive diagnosis of primary IgAN and/or for evaluating the risk for IgAN progression. However, despite numerous studies investigating underlying molecular mechanisms of IgAN and a number of proposed IgAN-specific serologic or urinary biomarkers (i.e., serum levels of Gd-IgA1, Gd-IgA1 specific autoantibodies, IgA-IgG immune complexes, urinary Gd-IgA1, CD89, CD71, miRNA) [72,73,74], it is important to be aware that all these proposed biomarkers have not been evaluated yet. Thus, their clinical value in predicting risk of IgAN and guiding clinical decision regarding IgAN diagnosis is currently not known.

Taken together, the pathogenesis of IgAN is a very complex process, in which several factors are involved, including the nature, glycosylation pattern of IgA, changes in the clearance of IgA from the circulation, and dysregulation of the IgA immune response.

## Figures and Tables

**Table 1:** Differences in the IgA form and subclass distribution in the serum and secretions in healthy human.

	SERUM IgA	MUCOSAL IgA
Production location	Plasmablast and plasma cells in the bone marrow, spleen, lymph nodes, tonsils [12]	Plasmablast and plasma cells located in the lamina propria of mucosal system (MALT, PP, ILF etc.) [20]
Production	≈ 1.2 g per day (adult human)	≈ 3.2 g per day
Form	75–90% monomeric, [12] 10–15% in polymeric form, [12] 1% bound in circulating immune complexes	Mucosa: 95% SIgA [12] lamina propria: predominantly pIgA, scarce mIgA
Subclass	85% IgA1, 12% IgA2 [12] 101 ± 26 (IgA1) and 14 ± 4 (IgA2) mg/kg body weight	% of IgA1 and IgA2 varies and depends on the mucosal area, i.e., tears, saliva, respiratory mucosa, vaginal, genital intestine (see Figure 1)
Function	Anti-inflammatory effects: binding of mIgA to FcαRI induce inhibitory effects and downregulates IgG-mediated phagocytosis, chemotaxis, bactericidal activity, oxidative burst activity, and cytokine release [21].	Bacteriostatic activity Barrier for microbiota (pathogens, and commensal bacteria), toxins from crossing the epithelial layer; neutralization of intracellular pathogens [13,18]
Clearance	Catabolized in liver, kidney, skin; a half-life ~5 days. Phagocytosis of IgA-Ag complex [22]	Secreted into lumen (excreted) Phagocytosis of IgA-Ag complex when in lamina propria or intraepithelial

**Legend**: MALT: mucosa associated lymphoid tissue; PP: payers patches; ILF: isolated lymphoid follicles. In the literature, differences in the amounts and percentages of mIgA/pIgA can be found, however, the amounts and percentages are stated mostly to get brief insight into the field.

**Table 2:** IgA receptors identified in humans and their function.

Receptor	Recognition Site (Ig Type, Form)	Cell Type Expressing the Receptor	Function	Ref
pIgR	J-chain (pIgA, pIgM)	Secretory epithelial cells in the intestine, salivary glands, bronchial mucosa, mammary glands, uterine	Transport of pIgA from lamina propria across the epithelial cells to secretions lumen, where it is released as SIgA (part of the receptor becomes part of SIgA; secretory component)	[30]
		Epithelial cells of biliary duct	Transport of serum pIgA into the bile (which excretes into intestinal lumen)	
DC-SIGN	*N*-/*O*-linked glycans (SIgA)	Dendritic cell Cell culture	Binding and internalizing SIgA	[31]
Dectin-1 Siglec-5	Cα1 and Cα2 (SIgA)	M cell	IgA-specific receptor on the apical surface that mediates the transepithelial transport to GALT	[28,32]
ASGPR	*O*-linked glycans on hinge region - terminal Gal or GalNAc of desialylated *O*-glycoproteins (free IgA1)	Hepatocytes	Clearance of IgA1 from the circulation and catabolic degradation (lysosomal catabolism)	[12,23,33,34]
		Monocytes	A mobile pool of the receptors, capable of reaching sites remote from the liver	
		Soluble form in circulation	Bind to free IgA1 in the circulation and transport to liver for uptake and degradation by hepatocytes [34]	
TfR TfR1 (CD71)	(IgA1; Monomeric better than polymeric)	Mesangial cells CD71 is expressed on a wide range of tissues TfR2 predominantly in the liver	Clearance of IgA from the circulation Binding of IgA1 to TfR1 depends on the size and glycosylation of IgA1 and can be inhibited by transferring	[24]
β-GalT1	Fc region	Mesangial cells	Clearance of IgA from the circulation	[25]
Leukocyte receptors				
SCR	Secretory component (SIgA, SC)	Eosinophils basophils	Generate respiratory burst and eosinophil degranulation, target killing, and release of proinflammatory cytokines and other mediators	[27,35]
FcRL4	Fc region (IgA)	Memory B cells in mucosal lymphoid tissue	Immune complex-dependent B cell regulation	[29]
Fcα/μR (CD351)	Fc region (IgA, IgM; Polymeric and immune complexes)	Mature B cells, macrophages Constitutively express	Endocytosis of IgA/IgM-coated microbes, phagocytosis higher affinity for IgM than IgA (10x)	[26]
FcαRI (CD89)	Fc region; CHα2 and CHα3, (immune complexes and pIgA better than monomeric SIgA only with lectin Mac-1)	Neutrophils, eosinophils, monocytes, Kupffer cells, macrophages, subpopulation of T and B cells, subset of DC, NK	Bifunctional receptor – the function depends on IgA ligand avidity: Anti-inflammatory: (free mIgA) inhibition of phagocytic activity, stimulate release of anti-inflammatory cytokines by myeloid cells Proinflammatory: (IgA + Ag complex receptor cross-linking): stimulation of phagocytosis, respiratory burst, release of ROS and proinflammatory cytokines, antigen presentation, antibody-dependent cellular cytotoxicity	[36,37]
		Soluble form of CD89 in circulation	Binds CD71 and induces TGase 2, which in turn is translocated to the mesangial plasma membrane allowing cell activation by IgA1-sCD89 complexes	

**LEGEND**: pIgR: polymeric immunoglobulin receptor; β-GalT1: β-1,4-galactosyltransferase1; ASGPR: asialoglycoprotein receptor; FcRL4: Fc receptor-like 4; TfR: transferrin receptor (CD71); SCR: secretory component receptor; FcαRI: Fc alpha receptor 1; GalNAc: *N*-acetylgalactosamine; GALT: gut associated lymphoid tissue; FcRL: Fc receptor-like.

**Table 3:** The prevalence of glomerular IgA deposition in kidney donors and necropsy cases.

Sample Size (Population)	Mesangial Deposition	Positive Cases	Clinical or Histological Features of Cases with IgA Deposits	Ref
Primary glomerular IgA deposition in individuals without clinical manifestation of renal disease				
510 kidney transplant cases (64 cadaveric and 446 living donors) (Japanese donors)	IgA IgA + C3	82/510 (16%) 16/82	IgA + C3 deposition was associated with mild degree of microhematuria, mesangial proliferation, and glomerular macrophage infiltration	[38]
756 autopsy cases (violent death) (Finland)	IgA IgA + C3b IgA + C1q IgA + IgG	52/756 (6.8%) 4/52 2/52 8/52	10/52 cases had morphological changes suggestive of renal disease	[39]
200 autopsy cases (violent death) (Singapore)	IgA IgA + C3b IgA + C1q IgA + IgG	8/200 (4%) 2/8 0/8 4/8	Histology revealed only minimal morphological alterations	[40]
Secondary glomerular IgA deposition				
250 consecutive autopsy cases (non-selected) (Germany)	IgA IgA + C3 IgA + C5 IgA + IgG(+)	12/250 (4.8%) 1/6 5/6 2/6	6/12 associated with liver cirrhosis 6/12 associated with endocarditis, bronchial asthma, cardiovascular disease, or neoplasia; IgA1+, IgA2 (+), SC (+)	[41]

**Table 4:** Heritability of serum Gd-IgA1 found in hereditary studies.

Sample Population	Controls Healthy Unrelated	Ancestry of Patients	Heritability (P Value)	Ref
89 adult IgAN patients vs. 266 blood relatives	150 adults	European	0.54 (0.0001)	[46]
63 adult IgAN patients vs. 32 first-degree relatives	44 adults	Chinese Asian	Yes nd	[48]
11 pediatric and 18 adult IgAN patients vs. 34 first-degree relatives	45 pediatric (European) 150 adult (European)	African American	0.74 (0.007)	[47]
14 pediatric IgAN patients vs. 25 first-degree relatives	51 pediatric 141 adults	European	0.76 (<0.05)	[49]
134 adult IgAN trios	638 adults	UK whites	0.387 (<0.05)	[45]
20 pediatric HSPN patients vs. 28 first-degree relatives	51 pediatric 141 adults	European	0.64 (<0.05)	[49]
27 monozygotic healthy female twin pairs 47 dizygotic healthy female twin pairs		European (UK)	0.84 0.46 (<0.05)	[50]

**Legend**: nd: not determined; HSPN: Henoch–Schönlein purpura nephritis. Increased levels of Gd-IgA1 in all above studies (except one [48]) were defined as levels that are 75% or 90% or 95% greater than the levels observed in geographically matched healthy controls.

**Table 5:** Genome-wide association studies (GWAS) and identified loci associated with IgAN.

GWAS	Sample Population	Ancestry of Patients	Identified Loci	IgAN Risk
[52]	914 cases vs. 5069 controls	European	6p–*HLA-B, DRB1, DQA, DQB*	nr
[53]	3144 cases vs. 2822 controls	Chinese, European	1q32-*CFHR1, CFHR3;* 22q12.2–*OSM, LIF, HORMAD2, MTMR3*; 6p21–*HLA-DRB1, HLA-DQB1, HLA-DPA1, HLA-DPB1, HLA-DPB2, TAP2, TAP1, PSMB8, PSMB9*	4–7%
[54]	4137 cases vs. 7734 controls	Chinese	8p23–DEFA, 17p13-*TNFSF13;* 22q12–*HORMAD2*	nr
[55]	7658 cases vs. 12,954 controls	European, East Asian	1p13-*VAV3*; 9q34–*CARD9*; 16p11-*ITGAM, ITGAX*; 8p23–DEFA; 6p21–*HLA-DQ-HLA-DR, TAP1–PSMB8* and *HLA-DP*; 1q32-*CFHR1, CFHR3;* 17p13-*TNFSF13;* 22q12–*HORMAD2*	5%
[56]	8313 cases vs. 19,680 controls	Chinese	3q27.3-*ST6GAL1;* 8p23–*DEFA*; 11p11.2-*ACCS*; 8q22.3-*ODF1-KLF10;* 16p11-*ITGAM, ITGAX*	1.7% 5%
[57]	2633 cases	European, East Asian	Xq24-*C1GALT1C1;* 7p21.3-*C1GALT1*	7% 2%
[58]	915 patients vs. 481 controls	Japanese	6p21–*HLA locus*; 12q12–*TSPAN8-PTPRR locus*	nr
[59]	498 patients vs. 893 controls	Koreans	10p15.1-*ANKRD16*	nr

**Table 6:** IgAN susceptibility loci discovered in genome-wide association studies (GWAS).

Locus	Gene	Function
1p13	*VAV3*	Chemokine signaling; NK, T cells, B cells, FcεRI, FcγR. VAV proteins are essential for adaptive immune function and NF-κB activation in B cells, i.e., a process that stimulates IgA production
1q32	*CFHR1, CFHR3*	Complement system; encode Factor H related peptides that modulate the activity of the alternative complement pathway. FHR1 competes with factor H for binding to surface-fixed C3b leading to activation of C3 convertase
6p21	*HLA-DQA1, HLA-DQB1, HLA-DRB1* *PSMB8, PSMB9, TAP1, TAP2*	MHC class II molecules critical for antigen processing and presentation and adaptive immunity Phagosome pathway
8p23	*DEFA1, DEFA3, DEFA4, DEFA5, DEFA6*	Innate immunity; antimicrobial peptides in mucosal defense; α-defensins 1,3,4 are synthesized in neutrophils, while α-defensins 5 and 6 are constitutively produced by the Paneth cells in the small intestine
8q22.3	*ODF1-KLF10*	Encodes a transcriptional repressor that acts as an effector of TGFβ signaling
9q34	*CARD9*	Innate immunity; NOD-like receptor signaling
11p11.2	*ACCS*	Encodes a1-aminocyclopropane-1-carboxylate synthase homologue that interact with a protein required for epithelial cell polarization and ciliogenesis
16p11	*ITGAM, ITGAX*	Encode leukocyte-specific α-integrins involved in the process of phagocytosis and regulation of IgA production
17p13	*TNFSF13*	Encode APRIL, a B cell stimulating cytokine induced by intestinal bacteria and promotes CD40-independent IgA class switching
22q12	*LIF, OSM, HORMAD2, MTMR3*	Cytokine-cytokine interaction; cytokine encoding genes expressed in mucosal tissues with immunomodulatory properties JAK-STAT signaling pathway
3q27.3	*ST6GAL1*	Encode enzyme ST3Gal-1 responsible for sialylation of Gal
7p21.3	*C1GALT1*	Encode enzyme C1GalT1 that catalyzes attachment of Gal to GalNAc
Xq24	*C1GALT1C1*	Encode chaperone Cosmc, required for the stability of C1GalT1 enzyme
